# Exposure of the Host-Associated Microbiome to Nutrient-Rich Conditions May Lead to Dysbiosis and Disease Development—an Evolutionary Perspective

**DOI:** 10.1128/mBio.00355-19

**Published:** 2019-05-14

**Authors:** Tim Lachnit, Thomas C. G. Bosch, Peter Deines

**Affiliations:** a Zoological Institute, Christian-Albrechts University Kiel, Kiel, Germany; University of Hawaii at Manoa

**Keywords:** dysbiosis, fasting, holobiont, host-microbe homeostasis, inflammatory disease, metaorganism, microbiome, overfeeding, starvation

## Abstract

Inflammatory diseases, such as inflammatory bowel diseases, are dramatically increasing worldwide, but an understanding of the underlying factors is lacking. We here present an ecoevolutionary perspective on the emergence of inflammatory diseases. We propose that adaptation has led to fine-tuned host-microbe interactions, which are maintained by secreted host metabolites nourishing the associated microbes. A constant elevation of nutrients in the gut environment leads to an increased activity and changed functionality of the microbiota, thus severely disturbing host-microbe interactions and leading to dysbiosis and disease development. In the past, starvation and pathogen infections, causing diarrhea, were common incidences that reset the gut bacterial community to its “human-specific-baseline.” However, these natural clearing mechanisms have been virtually eradicated in developed countries, allowing a constant uncontrolled growth of bacteria. This leads to an increase of bacterial products that stimulate the immune system and ultimately might initiate inflammatory reactions.

## Abstract (precis)

Inflammatory diseases, such as inflammatory bowel diseases, are dramatically increasing worldwide, but an understanding of the underlying factors is lacking. We here present an ecoevolutionary perspective on the emergence of inflammatory diseases.

## OPINION/HYPOTHESIS

The human body is associated with a huge diversity of different microbes colonizing skin, gut, teeth, and even epithelia of the lungs. Together with our microbes, we form a synergist relation, which is termed holobiont or metaorganism (1, 2). Disturbance of this host-microbe homeostasis can lead to dysbiosis (microbial imbalance on or inside the host) and/or disease development (3–5).

In the last several decades, inflammatory diseases, such as inflammatory bowel disease (IBD), are on the rise (6, 7). Different hypotheses have been put forward as potential explanations for this phenomenon, such as the hygiene hypothesis (8), the keystone-pathogen hypothesis (9), or genetic predisposition (10). Although there is evidence for different genetic predispositions that are associated with inflammatory diseases, they can explain only approximately 20% of the disease (11). Eighty percent is unexplained, and there is a lively debate on whether environmental factors could be a key trigger for the onset of disease and disease development (10).

It is well documented that inflammatory diseases are accompanied by changes in microbial density (12) or microbial community composition (13). However, comprehensive sequencing approaches have not yet led to the identification of a key pathogen, nor to the discovery of a specific pathobiome that is responsible for the disease (14). On the contrary, it is becoming more and more apparent that our associated microbiota is not as specific as we thought and that, even within the same individual, microbial community composition underlies strong temporal variability (13). So far, one common observation in the context of Western lifestyle and inflammatory disease is a reduction of microbial diversity (15). Although sequencing depth has dramatically increased by the development of new technologies, 16S rRNA amplicon sequencing only provides an overview of the associated bacterial members. Knowing that there is enormous variation within the genetic repertoire of bacteria featuring the same 16S rRNA sequence, researchers now search for insights into the interplay of bacteria within the gut by analyzing DNA (metagenomics), RNA (metatranscriptomics), proteins (metaproteomics), and metabolites (metabolomics) in order to elucidate their functional interactions within metaorganisms. Whether this approach will finally lead to the development of a more targeted approach to reconstitute natural host-microbe homeostasis in order to treat inflammatory disease is questionable as it is so far not clear if a disturbed microbiota is the cause or the consequence of inflammatory diseases (4).

Emerging infectious diseases are on the rise in not only the human population. Similar trends can be observed in the ocean over the last decade. Time series exist for only a few species such as corals and oysters, but both show the same overall pattern—an exponential rise in disease outbreaks (16, 17). Today, disease syndromes have been described for a variety of aquatic organisms of natural and cultured populations, including fish, seagrass, seaweeds, sponges, corals, and other invertebrates (18–21). As for inflammatory diseases in humans, in many cases the causative agents are unknown. It is evident, however, that a number of complex diseases in the aquatic environment are linked to a dysbiotic state of the microbiome (22–25). Anthropogenic impact, such as urbanization and global climate change, is one factor that can alter the microbiota of key habitat-forming species, such as corals and seaweeds, with potential ecological consequences (24, 25).

Here we introduce an alternative perspective and propose that host-secreted metabolites play a major role in maintaining symbiotic interactions by nourishing the associated microbes. Changes in the environment that interfere with this nutrient dependency of host-associated bacteria may severely disturb host-microbe interactions and thus lead to dysbiosis and disease development.

### Maintenance of host-microbe homeostasis.

To gain a better understanding of host-microbe interactions, we must consider that all eukaryotic life has evolved in an aquatic environment under the constant exposure to bacteria. This adaptation has formed a synergistic relation between the eukaryotic host and its associated microbes. No surprise that all epithelial surfaces are colonized by bacteria, ranging from marine algae (26, 27), plankton (28), corals and other marine invertebrates (29), freshwater polyps (30, 31), and marine vertebrates (32) to humans (33). Host-microbe interactions take place at epithelial surfaces that are exposed to both the bacterial colonizers and the surrounding environmental conditions. Surfaces of aquatic organisms, for example, are permanently subjected to a colonizer pool and have to regulate bacterial colonization to inhibit invasion and overgrowth by bacteria. Planktonic bacteria, however, are at certain times growth limited (34, 35), and it is reasonable to assume that it is advantageous to attach to surfaces. Evidence for this comes from bacterial ectosymbionts from marine nematodes, where the evolution of longitudinal cell division enhances bacterial attachment to its host (36). Whereas this represents a very specific case, it is known that already the attachment of bacterial cells to nonliving amorphous surfaces and the formation of biofilms lead to an increase in bacterial growth (37). The biofilm serves as a trap for nutrients from the surrounding water, providing shelter for bacterial cells against bacterivores, and facilitates degradation of complex compounds due to collaboration of diverse bacteria (38). Living attached to a nutrient-rich epithelial surface, featuring a carbohydrate source such as mucins or polysaccharides, should therefore constitute a fitness advantage to the bacteria. This colonization process must, however, be controlled by the host to limit the amount of surface-colonizing organisms and to select for nonpathogenic or even for beneficial bacteria. Indeed, there are several studies that have shown antibacterial effects of marine seaweeds (39–41) and many others regarding the innate immune system of invertebrates and vertebrates that allow them to sense and shape bacterial colonization (42, 43).

### Epithelial colonization: host secretion as nutrient resource.

**FIG 1:**
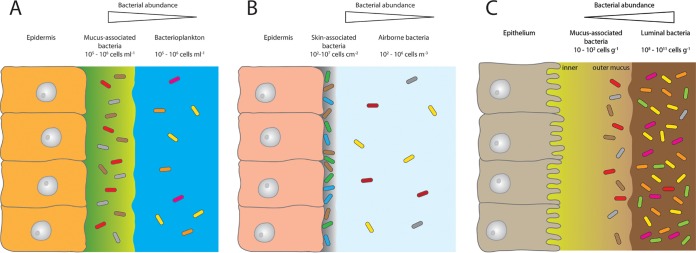
The relative abundance of bacteria associated with coral mucus (A), the human skin (B), and the mucosa of the gastrointestinal tract (C) in relation to the adjacent environments seawater, air, and the lumen of the gastrointestinal tract. Abundance data for the different environments were taken from references 19, 65, and [Bibr B80][Bibr B81][Bibr B87].

Under natural conditions, diverse bacterial communities cover epithelial surfaces of aquatic organisms. In environments that are low in dissolved organic matter (DOM) or particulate organic matter (POM), host-secreted metabolites can often present the only form of available nutrients (44). Examples include *Hydra*, which when kept in artificial *Hydra* medium (water plus salts) still features a complex host-associated bacterial community (30). Another example is corals, which have a mucus that is densely populated by a phylogenetically distinct bacterial community (e.g., references 2 and [Bibr B45][Bibr B46][Bibr B47]). Biomolecules released from the mucus may serve as potent chemoattractants for natural populations of coral reef bacteria (48). This finding supports the idea that the host is a major driver in providing access to resources for its surface-associated bacteria. In general, microbial abundances in coral mucus are roughly 1 order of magnitude higher than in the surrounding reef waters (49). Interestingly, when comparing the quantity of host surface-associated bacteria to that of the surrounding environment, we find the same pattern in aquatic and terrestrial organisms. For both, the abundance of surface-associated bacteria is always higher than in the surrounding environment (Fig. 1, corals [panel A] and human skin [panel B]). But there are also differences between the two systems. For terrestrial life, the rate of encountering bacteria is highly reduced as air bacterial counts make up only a fraction of reef water counts (Fig. 1, corals [panel A] versus human skin [panel B]). Further, the nutrient supply to, e.g., the skin environment from the air is almost zero. Skin-associated bacteria therefore have to live solely from secreted or surface-shed material. In the human gut, we find the exact opposite. Here bacterial abundances in the lumen exceed the mucus-associated bacteria by roughly a factor of 10^8^ (Fig. 1C).

### The evolutionary origin of two different microbial niches within the gut.

From an evolutionary perspective (see reference 50), the development of a gut environment changed the existing host-microbe relationships by introducing another habitat and food source for the bacteria. The invagination during gastrulation delocalized the epithelial surface from the outside into the inner part of an organism and separated it from environmental conditions in the water. Consequently, this led to dramatic environmental changes for the associated microbiota due to a restricted exchange with the surrounding water. For example, oxygen and nutrient supply from the surrounding water became limited. Instead, associated microbes were less exposed to the diverse mixture of bacterial colonizers, grazers, and phages than were the plankton community. This in turn limited the microbial colonization of the gut environment, which became dependent on the feeding behavior of the eukaryotic host. In general, the uptake of food does not only allocate new bacteria into the gut, but it also offers an alternative nutrient source for the gut microbiota and thus forms an additional habitat for bacteria. The development of a gut thus led to two different niches with distinct microbial populations: the gut mucus with its associated residential microbiota (which we here term “human-specific-baseline” microbiota) and the food that is being digested with its associated transient microbiota (luminal microbiome). While the growth of mucus-associated bacterial communities on the outside of organisms is restricted by host-secreted metabolites, mucus-associated bacteria in the gut are exposed to dynamic compositional changes of the digested/ingested food depending on: food consumption, food composition, frequency of feeding, digestive processing by the host, and also the enzymatic activity of the microbiota. Host-derived substances, such as bile acids, mucus, and urea, are important nutrient sources for the microbiota, with urea serving as a major nitrogen supply (51). These processes supply additional nutrients for mucus-associated bacteria and so continuously shape gut environmental conditions and affect microbial community composition (52–56).

### Maintaining homeostasis: natural recurrent clearing mechanisms.

In the evolutionary history of animals and humans, hunger was the default state. Finding food was always a costly endeavor. On evolutionary time scales, food availability was often limited and not only humans but also other organisms had to adapt to periods of food shortage (57, 58). There is increasing evidence that humans are well adapted to food shortages of up to 1 day. After that time, glycogen storage, which serves as an energy source, gets depleted and metabolism switches to gluconeogenesis (59). Already a longer nighttime fasting duration reduces the C-reactive protein (CRP) level when evening dinner contains less than 30% of the daily calorie needs (60). Fasting for even longer periods of time has been shown to have a huge impact on a variety of processes, such as changes in cellular pathways, prevention of disease development, and delay in aging not only in humans but also in animal models (59). Whereas fasting in lower eukaryotes leads to higher longevity and in rodents it protects against diabetes, cancer, heart disease, and neurodegeneration, in humans it helps to reduce obesity, hypertension, asthma, and rheumatoid arthritis (57). While such host metabolic changes induced by fasting are only partially understood, we here propose that an important factor that links fasting to our state of health is the effect of fasting on our gut microbiota. From many animals, including mice, alligators, pythons, and chickens, we know that fasting induces shifts in the gut microbiome (61). The same is true for hibernation, which has been shown to alter the diversity and composition of gut microbiota in ground squirrels (62). The authors could show that hibernation reduced populations of *Firmicutes* (which prefer polysaccharides) but increased populations of *Bacteroidetes* and *Verrucomicrobia*, capable of degrading mucin. This supports the idea that during a lack of nutrient availability, mucin glycans become the primary substrate for microbial metabolism during hibernation (63). In addition, fasting and hibernation not only lead to a changed microbiome composition but also suppress the immune system (64), thus increasing the tolerance of the host to its microbes.

In humans, food supply for the gut bacteria is also reduced during fasting, limiting uncontrolled growth of unspecific, non-mucus-adapted gut microbiota. Instead, the microbes that are favored are highly adapted to the gut environment in that they are able to degrade mucin or exist on host-derived secretions (65, 66). In the last century, food became permanently available and the amount of available food per capita increased by up to 10-fold in developed countries by the end of the century (67). These circumstances presented a new situation in which nutrients were continuously available not only for modern humans but also for their gut-associated bacteria.

While excessive food supply led to the loss of fasting events in the 20th century, sanitation conditions were improved at the same time in developed countries. The introduction of cleansing agents, purification of drinking water, and the invention of washing machines and refrigerators helped to reduce the risk of gastrointestinal pathogenic infections. This is in contrast to developing countries, where poor hygiene conditions and a higher risk for the population of contracting diarrheal diseases are found (68). Recurrent gastrointestinal pathogenic infections are accompanied by periods of diarrhea and so regularly reduce the population of the unspecific luminal microbiota. During human evolution, this “cleaning” effect might have been important for maintaining health as it provides the mucosally adapted microbiota with the opportunity to proliferate. While events of diarrheal infections have been shown to be followed by a rapid, reproducible, and reversible change in microbial community structure (69), the effect of diarrheal flushing on total bacterial abundance is not known. Although mild and short-term forms of diarrhea might be beneficial for human health, pathogen-induced diarrhea can also have severe consequences, especially in children, leading to significant morbidity and mortality. Not only diarrhea but also artificial cleansing of the gut by bowel preparation for colonoscopy has an impact on the gut microbial community. Due to this procedure, microbial load in the gut is decreased by about 30-fold (70) and bacterial diversity is reduced in the short term but restored after approximately 14 days (70–72). Another crucial novelty of the last decades is the use of antibiotics, which have become a key weapon to fight malignant bacteria. Despite their benefits in combating disease, they massively challenge our microbiome, leading to the loss of beneficial microbes (73), and likely impair the restoration of a healthy microbiome.

In general, the lack of natural cleaning mechanisms appears to result in a loss of the competitive advantage of the mucus-adapted bacteria while simultaneously favoring the non-mucus-adapted gut microbial bacteria that live on ingested food items of their host. During microbial breakdown of food residues, small molecules that can cross the epithelial border and get dispersed in the serum are produced. These molecules can either be harmful (e.g., uremic toxins) or beneficial (e.g., antioxidants) to the host (74). Depending on the food composition, the ratio of harmful to beneficial compounds can shift; so an increased protein fermentation, for example, increases the amounts of potential toxic compounds, such as ammonia, phenols, amines, indoles, and thiols (75). Interestingly, dietary shifts in the food quality in a Western-style diet (WSD), which is easily digestible by humans and microbes, being rich in sugars, fats, and proteins but having small amounts of fiber, can have adverse health effects, while diets rich in fiber are beneficial (76). There is initial evidence from a mouse model that the WSD alters the microbiome composition and so causes an increased penetrability of the mucus layer (77). By adding fibers to the diet, the authors were able to preserve the barrier function of the mucus (77). In contrast, a high dietary phosphate content of WSD can exacerbate intestinal inflammation in experimental colitis (78), pointing to an imbalance in the C/N/P ratio. The significance of C/N/P ratios in marine environments for the growth of organisms is well established (79), and it is reasonable to assume that it also plays a central role in the nutrient and microbiota homeostasis in the human gut.

### Excess feeding may disrupt the homeostasis and lead to an altered microbiome, resulting in disease development: the overfeeding hypothesis.

We here propose that overfeeding of the host-associated bacterial community, particularly with easily digestible, energy-dense, low-fiber-content foods, likely causes dysbiosis and the development of disease. Overfeeding uncouples natural host-microbe associations, leading to an increased activity and changed functionality of the associated microbiota. In previous times, starvation and also pathogen infections which lead to diarrhea were common incidences that may have helped to reset the gut microbial community to its “human-specific baseline.” However, these natural clearing mechanisms have been almost totally eradicated in developed countries, allowing an uncontrolled growth of bacteria which are not specifically adapted to the human host mucosal environment. This may lead to a changed composition and increase of bacterial by-products in the gut. Moreover, overfeeding by consuming a WSD may also impair the natural nutrient balance in the gut (C/N/P ratio), leading to an enhanced microbial degradation of the mucus barrier. Taken together, the reduction of the mucus barrier function and the increased release of bacterial by-products into the gut elevate nonself recognition of the host and stimulate the immune system. Additional nonself recognition and stimulation of the immune system anywhere in the body likely initiate an immune response, which might contribute to the development of complex disease, such as atopic dermatitis, asthma, or diabetes.
